# Visual perception of one’s own body under vestibular stimulation using biometric self-avatars in virtual reality

**DOI:** 10.1371/journal.pone.0213944

**Published:** 2019-03-18

**Authors:** Hans-Otto Karnath, Simone Claire Mölbert, Anna Katharina Klaner, Joachim Tesch, Katrin Elisabeth Giel, Hong Yu Wong, Betty J. Mohler

**Affiliations:** 1 Centre of Neurology, Division of Neuropsychology, Hertie-Institute for Clinical Brain Research, University of Tübingen, Tübingen, Germany; 2 Department of Psychology, University of South Carolina, Columbia, SC, United States of America; 3 Medical University Hospital Tübingen, Dept. of Psychosomatic Medicine and Psychotherapy, University of Tübingen, Tübingen, Germany; 4 Max Planck Institute for Biological Cybernetics, Tübingen, Germany; 5 Institute of Philosophy, University of Tübingen, Tübingen, Germany; 6 Technical University Darmstadt, Institute of Sports Science, Darmstadt, Germany; University of Zurich, SWITZERLAND

## Abstract

**Background and purpose:**

Vestibular input is projected to "multisensory (vestibular) cortex" where it converges with input from other sensory modalities. It has been assumed that this multisensory integration enables a continuous perception of state and presence of one’s own body. The present study thus asked whether or not vestibular stimulation may impact this perception.

**Methods:**

We used an immersive virtual reality setup to realistically manipulate the length of extremities of first person biometric avatars. Twenty-two healthy participants had to adjust arms and legs to their correct length from various start lengths before, during, and after vestibular stimulation.

**Results:**

Neither unilateral caloric nor galvanic vestibular stimulation had a modulating effect on the perceived size of own extremities.

**Conclusion:**

Our results suggest that vestibular stimulation does not directly influence the explicit somatosensory representation of our body. It is possible that in non-brain-damaged, healthy subjects, changes in whole body size perception are principally not mediated by vestibular information. Alternatively, visual feedback and/or memory may dominate multisensory integration and thereby override possibly existing modulations of body perception by vestibular stimulation. The present observations suggest that multisensory integration and not the processing of a single sensory input is the crucial mechanism in generating our body representation in relation to the external world.

## Introduction

The peripheral components of the vestibular system have a highly specialized function. Their rotation and acceleration information are responsible for the orientation of eyes as well as for head and postural control. Interestingly, neither neurophysiological findings in monkeys nor functional imaging, cortical stimulation and lesion analyses in humans reported the existence of an exclusive cortical representation of the vestibular input, i.e. a primary "vestibular cortex" [1,2]. Rather, a "multisensory (vestibular) cortex" has been described involving the superior temporal cortex, insula, retroinsular regions, and the temporo-parietal junction [1,3], with processing of vestibular input as only one component. Beyond vestibular information, these multisensory cortical areas integrate visual, optokinetic, and somatosensory signals [4–6]. More recent functional imaging studies with nociceptive, somatosensory, optokinetic, acoustic, vestibular, and even olfactory stimulation have confirmed the convergence of different sensory modalities and the multisensory character of these cortical regions [7–10]. Vestibular information thus is integrated in highly complex multimodal representations, whose exact roles are still being discussed.

Karnath and Dieterich [1] have suggested a fundamental role of the "multisensory (vestibular) cortex" in encoding higher order spatial representations that allow us to perceive and adjust the position and motion of our body relative to external space. Due to the close anatomical correspondence between these multisensory cortical areas and those areas associated with spatial neglect after brain damage [11], the authors argued that a disturbance of this conversion of multimodal sensory input may underlie the spatial bias in brain damaged patients with spatial neglect. In line with this assumption, asymmetric, unilateral vestibular stimulation was observed to have compensatory effects on the clinical signs of these patients [12–16]; under stimulation the neglect of contralateral information is temporarily reduced or even absent.

A further role of “multisensory (vestibular) cortex” has been suggested by Pfeiffer et al. [17], Ferrè and Haggard [2], as well as Lopez [18]. They argued that the multisensory convergence in these regions may impact self-representation, i.e. maintaining a continuous perception of state and presence of the body, relative to the environment. In line with the early work by Bonnier [19] and by Schilder [20] that suggested a modulation of our representation of the body (schéma) and of body parts by the activity of the vestibular system, they hypothesized that the interaction between vestibular signals and somatosensory inputs, in particular, might play a key role in distinguishing one’s own body from its momentary interactions with the world, in perceiving shape and size of the body, and in generating bodily self-consciousness and a reference for first-person perspective. In line with these assumptions, Ferrè and coworkers have observed increased somatosensory perceptual sensitivity [21] as well as increased threshold for detecting pain [22] immediately after left caloric vestibular stimulation (CVS), both ipsilaterally and contralaterally. Furthermore, studies observed that CVS temporarily increased the perceived length and width of the own hand [23] or decreased the perceived width of own thighs [24], suggesting that vestibular information is used to scale the internal representation of body segments. In a further study, Ferrè et al. [25] extended the experiments on bimodal interaction to the interaction with a third afferent input channel, namely with the visual system. In fact, they found evidence not only for separate but also combined vestibular and visual modulation of somatosensation.

The present investigation aimed to take a step further towards more complex bodily sensations. Following up on the observations by Lopez et al. [23] on the influence of vestibular input on perceived hand size, we here ask whether or not vestibular afferent input also impacts the perception of large parts of our body image. We made use of recent technical advances to investigate perception of one’s own body by using biometric self-avatars in virtual reality. To balance biases induced by specific stimulation methods, we used two different types of vestibular stimulation: subthreshold galvanic stimulation and caloric stimulation with ice water. Our methods allowed us to realistically manipulate the length of extremities of self-avatars in a well-controlled way under vestibular stimulation with different levels of side effects. In particular, we hypothesized that it is possible to influence the accuracy of length estimates of one’s own extremities by vestibular (galvanic or caloric) stimulation.

## Methods

### Participants

The number of participants was determined by a power analysis. Effect sizes were calculated based on previous data by Lopez et al. [23] on mean perceived length of the own hand obtained in healthy subjects under CVS in contrast to sham stimulation. Assuming a minimum improvement of 0.9 cm, the corresponding effect size $d=\frac{0.9}{1.69}=0.53$ indicates a medium effect according to Cohen. For our power calculation, we therefore assumed a medium effect size of f = 0.25 for the body side (left, right) x condition (pre, stimulation, post) interaction, alpha = .05, power = 0.80 [26], and an assumed correlation of r = .65 between repeated measures using GPower 3.1 software [27]. This calculation yielded a required sample size of 20 participants. Twenty-two healthy subjects (10 male; 20 right-handed; mean age 23 ± 3.7 years, range 18–30 years) who had no previous history of severe mental disorders, hearing impairments or vestibular problems finally participated in our study. They provided written informed consent to participate in the study which was conducted in accordance with the ethical guidelines from the Declaration of Helsinki and was approved by the ethics committee of the University Tübingen and the Medical Faculty Tübingen. One additional participant was excluded due to technical problems. One participant participated only in experiment 1 but not in experiment 2.

### Study design

**Fig 1:**
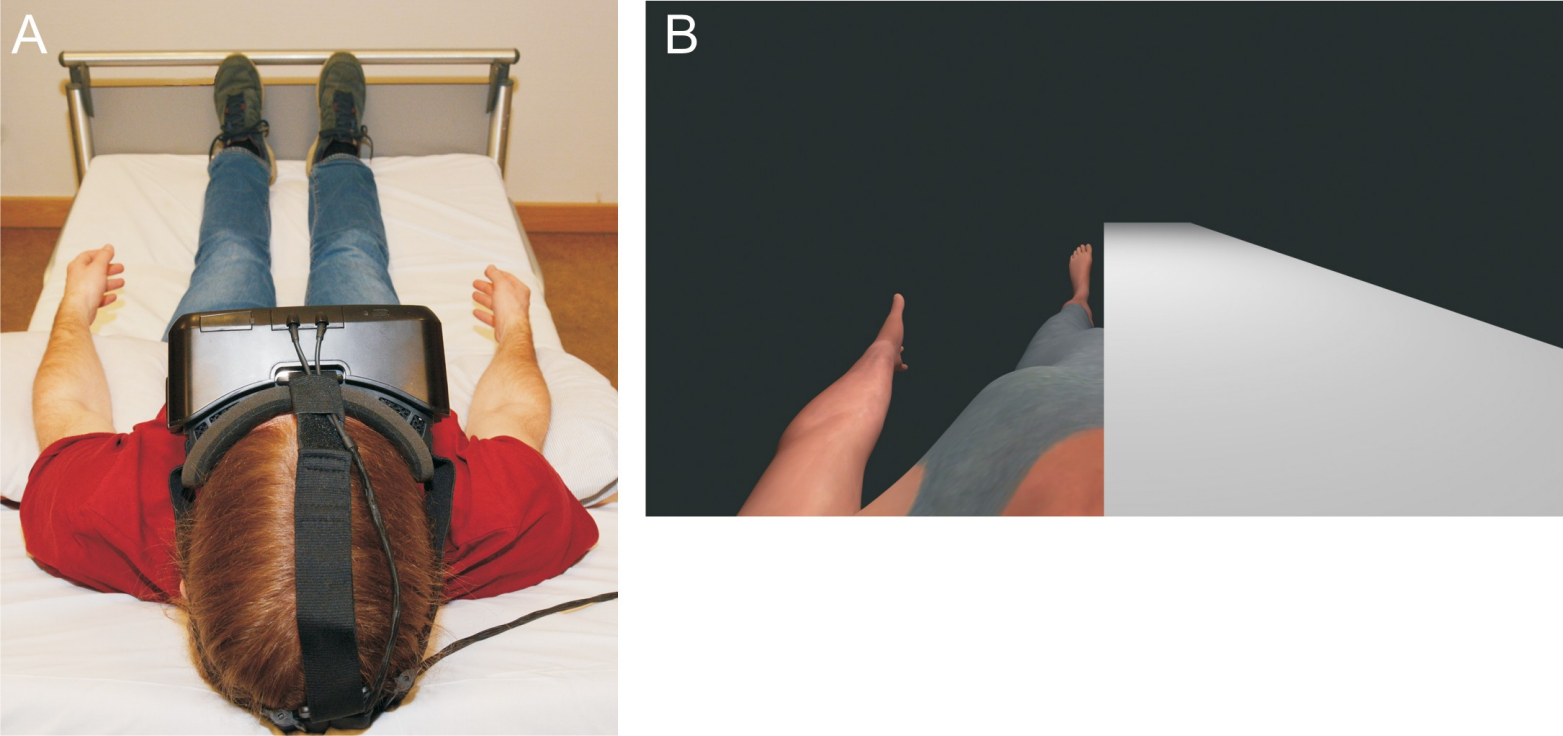
Study setup. Illustration of the study setting (*A*) and virtual reality setup (*B*).

The study comprised two experiments. In both experiments, participants lay in a bed in supine position with the head elevated and tilted ~30° forwards (Fig 1A). An immersive virtual reality (VR) setup provided participants with a first person biometric avatar of their body (Fig 1B) to assess visually perceived arm and leg length of the participants. At the beginning, the experimenter confirmed the inclusion criteria and explained the study procedure, then assessed self-reported height and weight and measured arm length (in T-pose) and leg length in the same body posture as implemented in the VR setup. All measures were taken at least twice to ensure validity. The participants’ task was to repeatedly adjust the arms and legs to their correct length from various start lengths. In both experiments, one out of three conditions was under vestibular stimulation. Experiment 1 used subthreshold left anodal galvanic vestibular stimulation (GVS); experiment 2 used caloric vestibular stimulation (CVS) of the left external auditory canal. After each stimulation and sham stimulation condition, perceived vertigo and side effects were assessed using a debriefing questionnaire. The two experiments were conducted in two separate sessions; the average time period between sessions was 6.3 ±4.7 days. Since it was important to have the GVS and CVS experiments analagous we opted to have an A-B-A rather than a counterbalanced design.

*Experiment 1 (GVS stimulation)*. The experiment took about 90 minutes and consisted of three conditions: baseline assessment before stimulation, assessment under subthreshold GVS (starting 15 minutes after termination of baseline assessment), and assessment under sham-‘stimulation’ after a 15 minutes break. The assessment under sham-‘stimulation’ was announced as second GVS condition, but the experimenter set the stimulation intensity to zero. Finally, the participants completed a short debriefing questionnaire, asking for strength and duration of vertigo and potential side effects (“Did you perceive vertigo during the stimulation (y/n)? / How strong was your vertigo on a scale from 0 (no vertigo) to 10 (very strong vertigo? / When did the vertigo start? / How long did the vertigo last? / Did you perceive nausea? / Did you notice any bodily changes along with the stimulation?”).

Left anodal subthreshold GVS was applied using a DC Stimulator Plus (neuroCare Group GmbH, Munich, Germany). Two rubber electrodes of 5x7cm size were placed over the left (anode) and right (cathode) mastoids; electrode gel was applied to reduce impedance. Before the experiment somatosensory thresholds were determined using a staircase procedure by Wilkinson et al. [28] that increased the applied current from 0.1 mA in 0.1 mA steps until the participant reported a tingling effect on the skin. The threshold was then verified by reducing the current by 0.3 mA and re-increasing it until the tingling effect re-occurred. For the experiment, a current of 95% of the individual ‘tingling’ threshold was applied, so that the subject did not consciously perceive any effect of the stimulation. A direct current signal was used that was ramped up over 5 seconds at the beginning of the stimulation and ramped down to zero at the end of the stimulation. The average threshold was M = 0.41mA ± 0.22mA, the stimulation was conducted at M = 0.38 mA ± 0.21 mA. GVS stimulation was applied continuously throughout the whole experimental block.

*Experiment 2 (CVS stimulation)*. The experiment took about 60 minutes. The experimenter confirmed intactness of the left tympanic membrane by otoscopic examination. Analogous to experiment 1, there were three conditions: baseline assessment before stimulation, assessment under CVS (starting about 15 minutes after termination of baseline assessment), and assessment after stimulation. The post-stimulation condition started 15 min after termination of the assessment under CVS, i.e. at a time when the caloric nystagmus had long decayed. Subsequently, the participants again completed the short debriefing questionnaire on perceived vertigo and side effects as well as another questionnaire asking for an evaluation of the VR setup, specifically for the perceived similarity between avatar and participant appearance in the virtual environment that were adapted from Piryankova et al. [29] and Mölbert et al. [30]. The exact questions of this second questionnaire are provided in Table 1.

**Table 1:** Overall evaluation of the VR setup at the end of the second session. M = mean, SD = standard deviation, Md = Median, IQR = Inter quartile range. N = 21.

How similar to you was the person that you saw through the glasses? (1 = not at all, 7 = very much)				
	M	SD	Md	IQR
Overall impression	4.48	1.5	4	2.5–4
Shape (e.g. proportions)	4.57	1.63	3	2.5–5
Appearance (like you or like a stranger?)	3.81	1.60	3	1.5–4
Arms	4.86	1.42	4	3–5
Legs	4.81	1.54	4	3–5
Torso	3.57	1.78	2	1–4
Did you have the impression that the setup represents you in a virtual environment?	Yes (8x), partly (4x), no (9x)			
Which strategies did you use to solve the task?	Relied on congruence with proprioception/interoception (11x), tried to remember length visually (4x), identification with visual impression (3x), comparison with other body parts (6x)			
Did any of the body parts look weird or even uncanny to you? If so, which?	Arms (5x), legs (3x), torso (5x), all (1x), none (7x)			
Do you have further comments or remarks on this study?	none			

CVS was applied by cold water irrigation of the left external auditory canal with 30 ml of ice water for 1 min. After stimulation, eye movements were observed using Frenzel glasses. In all subjects a brisk nystagmus was induced with the slow phase to the left side. Assessment procedures were started immediately after Frenzel glasses were put off and the VR head mounted display put on, i.e. ~15 sec after irrigation. Participants were instructed to notify the experimenter as soon as the induced vertigo had ceased. Since the assessment procedure under CVS typically lasted longer than the effects of irrigation, this condition provided trials in which subjects were under vertigo as well as trials after participants had reported that vertigo had ceased. For data analysis (see below) we only included trials under vertigo.

### VR setup for the assessment of perceived arm and leg lengths

The setup was created in the Unity game engine (4.6.3f1) using the Oculus Unity integration (0.4.4) and displayed through an Oculus Rift DK2 head mounted display. Within the setup, the participants’ bodies were represented through biometric avatars. The avatars were based on a statistic model of average male and female body shape that we derived from 96 body scans of participants aged 60+ from the CAESAR dataset [31]. The higher age focus was chosen to make the setup suitable for comparison with clinical populations. For each participant, we generated an avatar with semi-individualized body shape by adapting the average gender specific shape to the participant’s self-reported weight and height. Since we considered torso length a relevant factor we adapted the inseam until the participant’s pelvis height was reached and scaled the spine to compensate for potential differences to the desired height. For both genders, we used the same texture, i.e. outer appearance, in which the avatar was dressed with gray shorts and a top. The avatar's facial features were covered by a 3D model of the Oculus DK2 head mounted display.

The avatar was put in supine position and in A-pose (arms 45° from the torso) with upper body rotated upward by 20° and placed in an empty grey scenario without any visual cues but the body. The participants watched the scenario from the first person perspective of the avatar, simulating the avatar to be their virtual body (Fig 1B). To facilitate identification with the avatar, head orientation was tracked through the onboard inertial measurement unit of the VR glasses and animated by applying the measured rotation changes to the neck node in the 3D model. The remaining body was locked to an A-pose. To enable independent estimates for the left and right body side, grey covers were fixed on top of the avatar that could be enabled or disenabled so that either view on the left or right side of the virtual body could be blocked.

Participants were placed in the same lying position and instructed to lift their arms to match the visual input (Fig 1A). To avoid postural mismatch between the participant and the avatar, the participants’ arms were padded with cushions and participants were instructed to maintain this position throughout the entire experiment. In each trial, the participant’s avatar was set to have an arm or leg length that varied between ±40%, ±35%, ±30%, ±25%, ±20% of an average person’s arm/leg length. One side of the body was always masked to prevent symmetry strategies, which resulted in 20 trials per run to cover all starting positions and sides, and ten repetitions per limb with counterbalanced start lengths. In order to achieve the subjectively perceived ‘correct’ length of own arms and legs, the participants repeatedly adjusted arm and leg length by oral instruction (“shorter”, “longer”) to the experimenter. Morphing step size was set to 2.5% of the subject’s actual limb length. The experimenter only started the next trial when the participant had confirmed the ‘correct’ length. Participants did not receive any feedback on accuracy.

### Outcome parameters

To enable automatic measuring of the avatar and comparison to the participant’s actual dimensions, leg length was defined as distance between a vertex at the broadest point of the hips and plantar and arm length was defined as distance between the tuberculum majus and fingertip in T-pose. These distances were measured and recorded on the virtual avatar as well as on the participants. As outcome parameters, body perception indices (BPI) for all limbs were calculated according to the formula BPI = (estimated size/actual size) x 100. As we did not expect a priori any differential effects for length perception of arms and legs and relations between arm and leg estimates were stable at the baseline conditions of both experiments (see below), arm and leg length estimates were subsequently averaged for the left and the right side of the body, resulting in BPIs for the left and the right side of the body for all conditions. Effects of stimulation were analyzed using these aggregated BPIs for the left and right side.

### Data analysis

Experiment 1 and 2 were analyzed separately. First, a 3x2 ANOVA was performed on BPIs with condition (pre, stimulation, post) and body side (left, right) as within subject factors. For the CVS stimulation condition, we only included trials for which the participants reported vertigo, resulting in only 7.6 ± 1.9 (range 4–10) trials instead of 20, and an imbalance in the presented starting lengths of the avatar’s arms/legs towards more long starting lengths on the right body side and more short starting lengths on the left. Since some of the variables did not meet all assumptions for parametric testing, we also explored the respective comparisons using non-parametric tests. This did not change the results. To further explore significant effects, we calculated post-hoc t-tests. Non-significant effects of condition were further explored with equivalence tests for the pre-vs.stimulation comparisons. Here, we set the smallest effect size of interest (SESOI) to the effect size we had 80% power to detect. Equivalence tests were calculated in R using the TOSTpaired function of the TOSTER package [32,33].

## Results

In the debriefing questionnaire asking for feedback on the VR setup, none of the participants reported any strange perceptions regarding the virtual body. The rated similarity between the avatar as a whole (“overall impression”) and the participant’s body corresponded to previously observed ratings using height and weight matched avatars with an average body shape and a photorealistic texture in a virtual mirror scenario [29]. Answer summaries are presented in Table 1.

The average height of the participants was M = 1.74 m ± 0.09m, their average weight was M = 68.3 kg ± 13.3 kg. Average arm length was M = 0.68 m ± 0.06 m, average leg length M = 0.85 m ± 0.08 m. In the baseline condition of both experiments, participants overestimated their limb lengths (experiment 1: left arm: + 21.7% ±16.1; left leg: + 7% ±17.3; right arm: + 24.7% ±16.1; right leg: + 7.3% ±16.7, experiment 2: left arm: + 19.4% ±19.4; left leg: + 7% ±18.9; right arm: + 20.6% ±16.4; right leg: + 6.9% ±18.3). Pairwise t-tests revealed that overestimation was consistently more pronounced for the arms than for the legs (all p < .014, two-sided). While there was a stronger overestimation for the right side compared to the left (+ 1.6%) in experiment 1 (t(21) = -2.51, p = .02, d = 0.16), both sides of the body were equally overestimated in experiment 2 (t(20) = 0.78, p = .45, d = 0.04).

**Fig 2:**
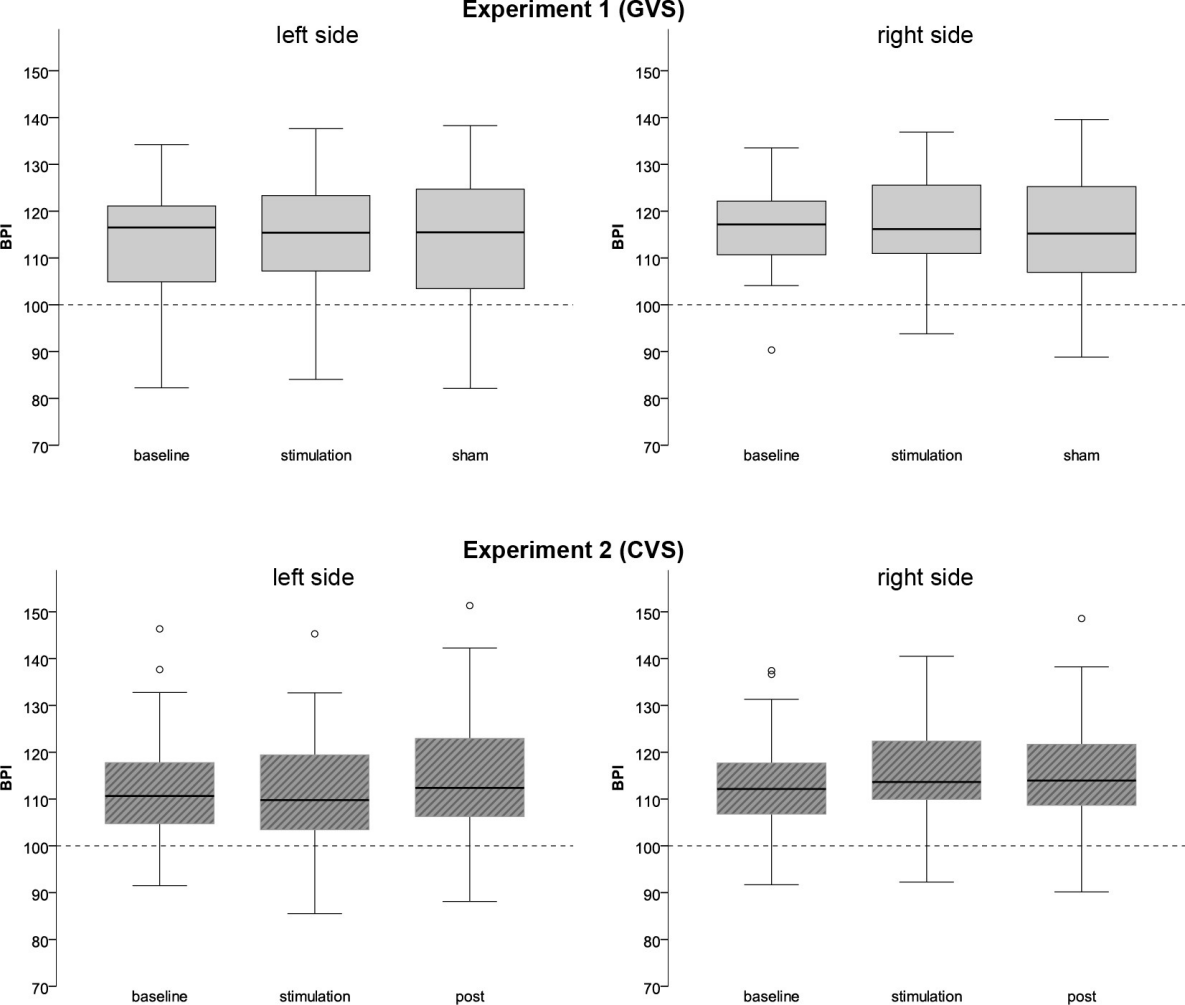
Body perception results. Boxplots of aggregated body perception indices (BPI; estimated/actual length * 100) for the left and right body side at baseline, under vestibular stimulation and at sham/post-assessment. BPI > 100 indicates overestimation, BPI < 100 indicates underestimation. There was no significant change in BPI due to vestibular stimulation in any of the experiments. Experiment 1 (left anodal subthreshold galvanic vestibular stimulation; GVS) is depicted in the top row; experiment 2 (left caloric vestibular stimulation with cold water; CVS) is depicted in the bottom row.

*Experiment 1 (GVS stimulation)*. Five participants (23%) reported slight subjective vertigo under GVS stimulation, on average of strength 2 ± 1 on a rating scale from 0 (no vertigo) to 10 (very strong vertigo). Three of these plus five other participants (38% of the sample) also reported body related changes, such as headache, tingling sensations, or subjective difficulties in task performance. Other reported bodily changes were increased alertness, subjective rotation and subjective difficulties in size estimation. Under sham stimulation, two participants (9%) experienced vertigo and three participants experienced body related changes (heat sensations, dizzy vision, sinuses pressure). No participant experienced vertigo in both conditions. Table 2 and Fig 2 illustrate the observed BPIs for the participants’ left and right body side. The 3x2 ANOVA on BPIs with condition (pre, stimulation, post) and body side (left, right) as within subject factors revealed a significant main effect for body side (F(1,21) = 7.58, p = .01, Eta^2^ = 0.27), indicating that the length of the extremities on the right body side was overestimated slightly more than on the left. The interaction condition*side (F(2,20) = 0.13, p = .88) as well as the main effect for condition (F(1.53,20) = 0.39, p = .62) were not significant. Equivalence tests for the pre-vs.stimulation comparison were significant (left: t(21) = 2.47, p = .011; right: t(21) = 2.38, p = .013; alpha = 0.05, equivalence bounds ±0.62), suggesting that estimates before and under stimulation were equivalent within the SESOI.

**Table 2:** Means (M) and standard deviations (SD) for the aggregated body perception index (BPI; estimated/actual length * 100) in each experimental condition. BPI > 100 indicates overestimation, BPI < 100 indicates underestimation. GVS = left anodal subthreshold galvanic vestibular stimulation; CVS = caloric vestibular stimulation (cold water irrigation to the left auditory canal).

	BPI left		BPI right	
	M	SD	M	SD
**Experiment 1** *(N = 22 subjects)*				
Pre	114.4	11.3	116.0	9.8
GVS	115.0	13.4	116.7	11.8
GVS sham	114.1	14.3	115.5	13.2
**Experiment 2** *(N = 21 subjects)*				
Pre	113.2	13.9	113.8	12.0
CVS	111.3	14.2	116.2	13.3
Post	115.1	15.8	115.8	14.4

*Experiment 2 (CVS stimulation)*. Under CVS stimulation, all participants reported strong vertigo, on average of strength 7.95 ± 1.47 on a rating scale from 0 (no vertigo) to 10 (very strong vertigo). Ten participants (47%) reported subjective changes of their bodily experience, such as headache, sweat, pressure in the stimulated ear; thirteen participants (62%) reported nausea. On average, the induced vertigo lasted for 3.05 ± 1.34 minutes. Table 2 and Fig 2 illustrate the observed BPIs for the participants’ left and right body side. The 3x2 ANOVA on BPIs with condition (pre, stimulation, post) and body side (left, right) as within subject factors revealed a significant main effect of side (F(1,20) = 10.03, p = .005, Eta^2^ = 0.33), no significant main effect for condition (F(2,19) = 2.08, p = .14), but a significant interaction effect side*condition (F(1.26,19) = 21.88, p < .001, Eta^2^ = 0.52). Post-hoc t-tests revealed that this was due to stronger overestimation for the right side of the body under CVS stimulation (about 5% more than for the left side, t(20) = -5.09, p < .001, d = 0.35), while there was no such side difference before and after stimulation (pre, post; all p>.30). Post-hoc t-tests between the three conditions (pre, stimulation, post) performed separately for the left and the right body side revealed no results that survived correction for multiple testing (all p> 0.006). Equivalence tests for the pre-vs.-stimulation comparison supported these observations: The equivalence test for the left side suggested equivalent estimates before and under stimulation (t(20) = 4.89, p < .001; alpha = 0.05, equivalence bounds ±0.64). For the right side, the test was non-significant, suggesting that estimates before and under stimulation might have differed (t(20) = 0.83, p = .209; alpha = 0.05, equivalence bounds ±0.64).

## Discussion

We used biometric self-avatars in virtual reality to investigate perception of one’s own body image under vestibular stimulation. Our method allowed us to realistically manipulate the length of extremities of semi-individualized self-avatars and investigate subject’s body perception from a first person perspective. Along the assumptions on the role of vestibular input on body perception [2,17,18], one might have expected that it is possible to influence the perception of large parts of one’s own body by vestibular stimulation. However, neither unilateral vestibular stimulation of the horizontal semicircular canal by caloric irrigation of one ear nor of the whole vestibular nerve by galvanic stimulation over the mastoid showed a modulating effect on the perceived size of own extremities. Subjects showed undisturbed body perception despite a clearly induced tonic imbalance in the bilateral vestibular system, provoking − under CVS − identical vestibular symptoms as observed with a unilateral lesion of the peripheral vestibular system, namely vestibular nystagmus and vertigo.

The present findings are in line with the observation by Ferrè et al. [34] that hand representation (perceived length and width) was not influenced by GVS. In their experiment, one hand of the healthy subjects was occluded and participants used a stick to indicate with the other hand the perceived location of verbally-identified landmarks on their occluded hand. By using the same behavioral task but a much stronger vestibular stimulus, namely CVS, Lopez et al. [23] observed that CVS temporarily increased the perceived length and width of the own hand. This discrepancy between the studies by Ferrè et al. [34] and by Lopez et al. [23] could have been accounted for by the different types of vestibular stimulation. The present study thus used both GVS as well as CVS. Interestingly, neither of the two types of stimulation had an impact on the subject’s perception of shape of own extremities. The present observations thus dissociate from those reported by Lopez et al. [23], even though the type of vestibular stimulation was comparable.

A possible explanation for this discrepancy might be that in neurologically healthy subjects changes in size preception of (large parts of) the body are not mediated by vestibular information. Indeed, only few observations in only very specific conditions have been reported where changes in vestibular input had an impact on size preception of (large parts of) the body. For example, under microgravity conditions, subjects may experience inversion illusions. One form of inversion illusion involves a change in both body size and shape: subjects experience their bodies retracting downwards (like a telescope) toward their feet and then being inverted [35]. There is also evidence of vestibular modulation of body shape with CVS in amputees and paraplegics. Temporary phantom limbs were induced in amputees and paraplegics who had not experienced phantom limbs previously [36,37].

Another possible explanation for the present findings might be a specific role of visual input for the perception of one’s own extremities. It has been suggested that vision can dominate multisensory processing and that knowledge about the structural organization of the body is primarily derived from vision [38,39]. An obvious experimental difference between the present study and the study by Lopez et al. [23] is that the subjects in the latter investigation were blindfolded. Judgements on perceived length of external objects touching the paticipant’s hand (their experiment 1) and of perceived length and width of the hand (their experiment 2) with and without the influence of CVS were based on tactile information only; vision was excluded. In contrast, the participants of the present study had to explicitly look at their experimentally manipulated extremities to adjust their lengths. This could mean that in healthy subjects visual feedback on arms and legs may override possibly existing modulations of body perception by vestibular stimulation and that such modulations can only be detected if vision is excluded. Indeed, the microgravity inversion illusion described above was only present in the absence of vision [35]. Further observations strengthen this interpretation.

In healthy subjects, it is possible to provoke neglect-like phenomena under vestibular stimulation. Beyond a nystagmus, unilateral vestibular stimulation induces a tonic shift of the average horizontal eye position with the nystagmus [40,41]. More interestingly, vestibular stimulation in complete darkness even induces a bias of the exploratory scan path in healthy subjects that resembles the spontaneous, asymmetrical, spatially biased exploratory behaviour of neglect patients [14], leading to ‘neglect’ of one side of the surrounding scene. A further consequence of unilateral vestibular stimulation in healthy subjects is a tonic bias of head orientation around the yaw axis [42]. This head orientation bias resembles the tonic head position bias that is observed in neurological patients suffering from spatial neglect [43]. However, all these effects evoked in healthy subjects under vestibular stimulation are immediately suppressed if the room light is switched on, i.e. if visual feedback of the surroundings and of own body orientation is provided. In good correspondence, vestibular tone imbalance due to a unilateral defect of the peripheral vestibular organ in neurological patients without brain damage does not cause spatial neglect if vision is provided under normal lighting conditions [44]. Vsion thus is able to override existing modulations by vestibular stimulation.

As for neglect-like phenomena [14,42], it is possible to also provoke derealisation phenomena in healthy subjects under vestibular stimulation. Mild symptoms pointing to an abnormal sense of familiarity with one’s own body [45] or the induction of the “rubber hand illusion” [46,47] can be provoked. However, and again in parallel with what has been observed in healthy subjects for neglect-like phenomena (see above), it has never been reported that vestibular stimulation in healthy subjects led to the full-blown phenomena of asomatognosia and/or somatoparaphrenia seen in right hemisphere stroke patients with anosognosia for hemiparesis/-plegia. Such patients not only deny the weakness of their paretic/plegic limb(s) but also show a disturbed sense of ownership; they experience their limb(s) as not belonging to them or as missing (asomatognosia), or may even attribute them to other persons (somatoparaphrenia) [48]. Interestingly, asymmetric, unilateral vestibular stimulation in such patients has transitory compensatory effects on these clinical signs [49–52]. If in healthy subjects only mild symptoms [45] but not the full-blown phenomena seen in neurological patients can be provoked under vestibular stimulation, it is probably the visual feedback from the environment and one’s own body that prevents disintegration of the normal unity of the self and the environment in non-brain-damaged subjects.

A further factor that might have contributed to keep visually perceived limb length stable under vestibular stimulation in the present experiments is visual memory. In a study of hand size estimation, estimates based on proprioceptive information indicated a distorted model of the hand, but still subjects were accurate in a visual matching task which required subjects to pick out the hand closest in shape to theirs [53]. These observations suggest that there may be a strong visual memory of body shape and size which may dominate and even overwrite other sources of afferent information.

Beyond the possibility that vision and/or visual memory has overriden possibly existing modulations by vestibular stimulation, one might also speculate about a possible role of embodiment. Embodiment, i.e. the induction of ownership over an artificial body or body part by synchronous visuotactile stimulation has been demonstrated to alter bodily experience [54–56]. The illusory ownership is achieved by observing from a first-person perspective how the virtual body (part) is being touched while a synchronous input is perceived on the actual body (part). The present study did not aim to induce a particular status of or to manipulate embodiment; the purpose of the use of a semi-individualized avatar was simply to create a sufficient self-specificity of the stimuli to allow an easy and straightforward manipulation of the length of the presented extremities. Nevertheless, we have measured the perceived similarity between avatar and participant appearance in the virtual environment at the end of the study; it was 4.5 on a scale from 1 to 7 and 57% of participants even reported to feel embodied with the avatar. Since one can expect that completely unrealistic avatars would have been rated with null similarity and null embodiment, we conclude that our setup had high self-specificity and presented the participants with plausible bodies. This reasonable perceived similarity achieved without synchronous visuotactile stimulation probably was due to the realistic presentation of the virtual body in the same location and posture as the physical body [55]. Still, it is possible that during the different experimental conditions of our study different embodiment levels might have existed that were not identical to the one measured at the end of the study and that this circumstance might have contributed to the present findings. Although the presentation of the biometric avatar was kept constant throughout all experimental conditions, we cannot exclude this possibility but would like to note that embodiment was not a prerequisite for meaningful data for the purpose of our study. Nevertheless, future investigations should explicitly test the influence of embodiment on body size perception with vestibular stimulation.

One effect of body size estimation observed in the present study not discussed so far was the observations that the length of own extremities was overestimated slightly more on the right body side than on the left. While this was the case in all three experimental conditions of our first experiment, this overestimation was observed only for the CVS condition but not the two contrasting conditions in the second experiment. Thus, the effect was not evoked by vestibular stimulation, as it occurred in conditions with as well as without vestibular stimulation. It rather appears to represent a more general attitude in healthy subjects which, however, is not reliable and apparently of only small magnitude. Differences between left and right body side have previously been reported for, e.g., somatosensory perceptual sensitivity [21] or perceived extent of reach [57], but without allowing a clear overall picture so far. Future studies are needed to further investigate this issue and clarify possible underlying mechanisms.

To conclude, our present experiment did not find evidence for a direct influence of vestibular stimulation on the explicit somatosensory representation of large parts of our body. This conclusion, of course, needs further empirical validation, e.g., by using different experimental setups and methodological approaches. Nevertheless, it is surprising in so far that current theoretical accounts of vestibular processing have assumed such a role for the representation of our body relative to its environment [2,17,18]. It is possible that in non-brain-damaged, healthy subjects changes in size preception of (large parts of) the body are principally not mediated by vestibular information. Alternatively, visual feedback and/or memory may override possibly existing modulations of body perception by vestibular stimulation and thus may only be detected if vision is excluded. Further studies therefore are needed to explore the influence of such factors for our perception of shape and size of the body and for our ability to discriminate one’s own body from its environment. The present observations argue for the view that multisensory integration and not the processing of a single sensory input is the crucial mechanism in generating body representation in relation to the external world. In other words, body representation appears to be sufficiently robust to compensate for (odd) vestibular signals. Redundant and robust body representation could be the key principle in ensuring a stable bodily self-consciousness and perception of the external world.
